# Comparison of COVID-19 Induced Respiratory Failure and Typical ARDS: Similarities and Differences

**DOI:** 10.3389/fmed.2022.829771

**Published:** 2022-05-27

**Authors:** Sen Lu, Xiaobo Huang, Rongan Liu, Yunping Lan, Yu Lei, Fan Zeng, Xuemei Tang, Hongli He

**Affiliations:** Department of Intensive Care Unit, Sichuan Provincial People's Hospital, University of Electronic Science and Technology of China, Sichuan Provincial Key Laboratory for Human Disease Gene Study, Chengdu, China

**Keywords:** COVID-19, ARDS, pathogenesis, pathophysiology, treatment

## Abstract

Coronavirus disease 2019 (COVID-19) is a predominantly respiratory infectious disease caused by novel coronavirus infection (SARS-CoV-2), respiratory failure is the main clinical manifestation and the leading cause of death. Even though it can meet the acute respiratory distress syndrome (ARDS) Berlin definition, only some clinical features of COVID-19 are consistent with typical ARDS, and which has its own peculiar phenotypes. When compared with typical ARDS, in addition to the typical diffuse alveolar injury, COVID-19 has unique pathological and pathophysiological features, such as endothelial injury, extensive microthrombus, and pulmonary capillary hyperplasia. The clinical features of patients with respiratory failure caused by COVID-19 are heterogeneous and can be generally divided into two phenotypes: progressive respiratory distress and unique “silent hypoxemia”. The “H-type” characteristics of reduced lung volume, decreased lung compliance, and unmatched ventilator-perfusion ratio. While some patients may have close to normal lung compliance, that is “L-type”. Identifying the exact phenotype in whom are suffered with COVID-19 is crucial to guide clinicians to adopt appropriate treatment strategies. This review discussed the similarities and differences in the pathogenesis, pathophysiology, clinical features and treatment strategies of COVID-19 induced acute respiratory failure and typical ARDS.

## Introduction

Coronavirus disease 2019 (COVID-19) is a predominantly respiratory infectious disease caused by novel Coronavirus infection (SARS-COV-2), which mainly affected the respiratory system, other organs were less involved. The clinical presentation of COVID-19 is highly heterogeneous, ranging from asymptomatic to severe respiratory failure (1). People with respiratory failure caused by COVID-19 could deteriorate to requiring invasive mechanical ventilation or death (2).

In theory, COVID-19 associated respiratory failure belongs to acute respiratory distress syndrome (ARDS) according to the Berlin definition (3). The respiratory symptoms of COVID-19 patients occur around 1 week, most patients present bilateral opacities in chest imaging and these patients always have severe hypoxemia needing respiratory support (2, 4). Physicians commonly use respiratory support strategies for ARDS to treat COVID-19 patients (2), however, some patients didn't response well and the mortality rate was as high as 61% (5). Besides, some researchers found that some patients presented unique “silent hypoxemia” without significant respiratory distress (6), or COVID-19 patients with respiratory failure present intriguingly high compliance (7), which are not consistent to typical ARDS. These differences between COVID-19 related acute respiratory failure and typical ARDS raised a lot of debates that whether COVID-19 pneumonia is ARDS or not? (8).

**Figure 1:**
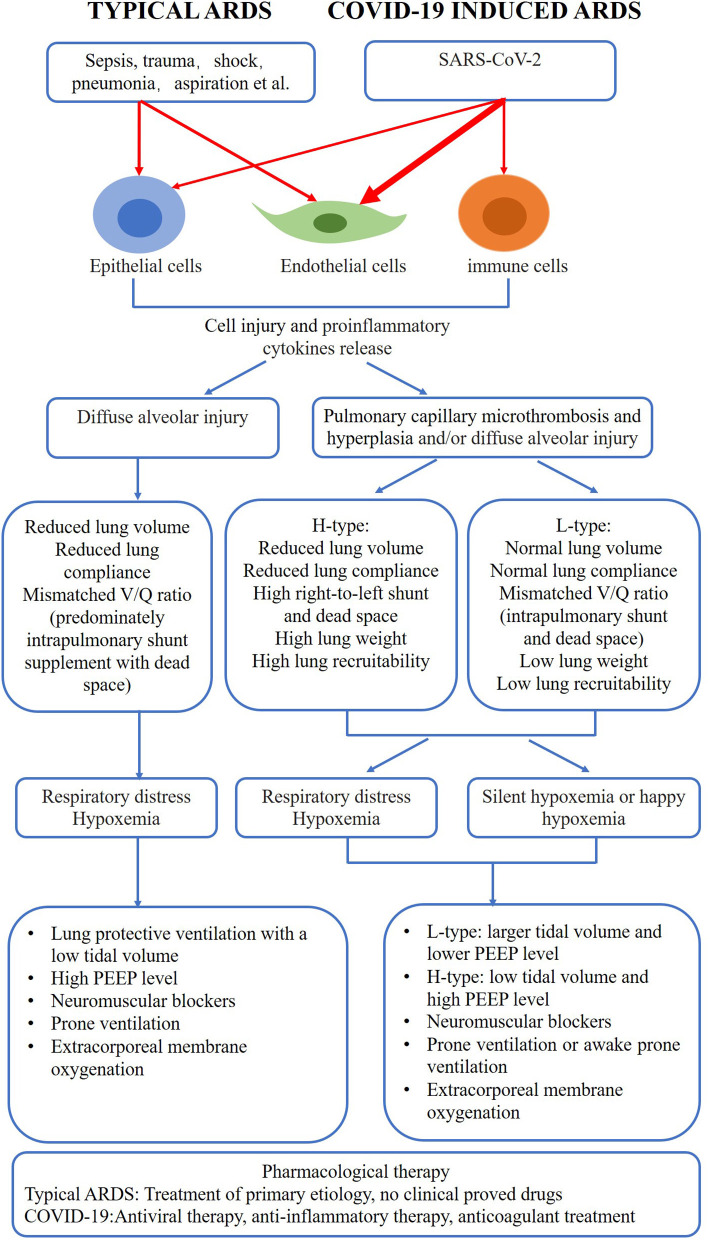
Comparison of COVID-19 induced respiratory failure and typical ARDS. COVID-19, coronavirus disease 2019; ARDS, acute respiratory distress syndrome.

The COVID-19 pandemic is still serious around the world. Scientists have made a lot of efforts to study this disease during the past 2 years, and more and more evidences about the pathogenesis and treatment have emerged. Therefore, a review which details key similarities and differences in the pathogenesis, pathophysiology and clinical features between COVID-19 related respiratory failure and typical ARDS is useful in understanding this disease and guiding the clinicians to alter strategies in care for COVID-19 patients (Figure 1).

## Pathogenesis and Pathology of COVID-19 and Typical ARDS

### Pathogenesis and Pathology of ARDS

The pathogenesis of ARDS has not been known very well. It is widely believed that lung parenchyma cell and several kinds of immune cells are involved in uncontrolled inflammatory response, which leads to the development of ARDS. Several clinical disease or disorders can lead to ARDS, such as sepsis, bacterial or viral pneumonia, trauma, aspiration pneumonia, shock and pancreatitis. On the one hand, the above insults can directly cause damage to lung parenchymal cells; one the other hand, the resident alveolar macrophages and dendritic cell are activated, leading to a robust release of proinflammatory cytokines and chemokines that promote the accumulation of neutrophils and monocytes in lung. Activated immune cells along with the injured epithelial and endothelial cells induce a further injury to promote the sustaining inflammation and tissue injury. The resultant injury leads to loss of barrier function, and accumulation of protein-rich edema fluid within the interstitium and alveolus, finally cause respiratory failure (9, 10).

The typical pathological feature of ARDS was diffuse alveolar injury (DAD), including hyaline membranes, edema, cell necrosis, or fibrosis, which was described by Katzenstein et al. in 1976 (11). ARDS is not a single disease but a set of clinical syndromes caused by multiple etiologies. In consequence, not all ARDS patients present typical DAD pathologically due to the different etiologies and pathogenic mechanisms (9, 10).

### Pathogenesis and Pathology of COVID-19

Contrast to the typical ARDS, the pathogen of COVID-19 is clear, caused by SARS-CoV-2, which is a beta-coronavirus that bind the angiotensin-converting enzyme-related carboxypeptidase (ACE2) receptor through the viral structural spike (S) protein to gain entry to cells. ACE2 receptor are widely distributed in alveolar epithelial cells (II), bronchial epithelial cells, vascular endothelial cells, small intestinal epithelial cells and several kinds of immune cells, including monocytes, macrophages and dendritic cells (12, 13). It means that SARS-CoV-2 can directly result to lower respiratory infection and induce inflammatory response by attacking immune cells in the lung.

Due to the abundance of blood vessels in lung tissues, SARS-CoV-2 infection can directly cause extensive damage to pulmonary vascular endothelium, airway and alveolar epithelium also show varying degrees of damage. The activation of stimulated endothelial cells further mediates the rolling, adhesion, migration of inflammatory cells, and activates inflammatory cascades and coagulation. Eventually, these processes will cause barrier damage, diffusion dysfunction and coagulation activation.

The pathological manifestations of COVID-19 include pulmonary edema, fibrinous exudation and inflammatory cell infiltration, alveolar septal vascular congestion, edema, vascular thrombi with focal intraparenchymal hemorrhage, and hemorrhagic infarction (14). DAD was reported in 67% to 100% of these autopsy patients (15, 16), which are compatible with typical ARDS. However, due to the serious damage to the endothelium, the COVID-19 patients showed distinctive vascular features, such as microthrombosis and hyperplasia. Varga et al. (17) reported that SARS-CoV-2 infection facilitates the induction of endotheliitis in several organs as a direct consequence of viral involvement (as noted with presence of viral bodies) and of the host inflammatory response. Ackermann and colleagues (15) also found that the lungs from patients who died of COVID-19 showed unique vascular characteristics. In addition to severe endothelial injury with the presence of intracellular virus and disrupted cell membranes, the incidence of pulmonary capillary microthrombi in COVID-19 patients was 9 times higher than that in influenza patients, accompanied by pulmonary capillary hyperplasia through a mechanism of intussusceptive angiogenesis. In an autopsy study of 12 consecutive patients who died of COVID-19, the incidence of deep venous thrombosis was as high as 58%, one third of the patients had a pulmonary embolism as the direct cause of death (16). Thus, these data suggest that the predominant vascular changes including endothelial cell injury, pulmonary capillary microthrombosis and hyperplasia are the main features of the patients with COVID-19, which lead to a different pathophysiological process and different response to treatment when compared with ARDS. Although most of the patients showed DAD, however, these data come from autopsies of patients who died from COVID-19. DAD is probably a result of late stage of this disease and not an early marker.

## Pathophysiology of COVID-19 and Typical ARDS

### Pathophysiology of Typical ARDS

ARDS is characterized by extensive injury of alveolar epithelial cells, pulmonary capillary endothelial cells and infiltration of inflammatory cells, resulting in high permeability pulmonary edema and diffuse inflammatory response. With the exudation of large amount of protein-rich fluid in the alveoli and lung interstitium, and the decrease of alveolar surfactant, a large number of alveoli collapsed and the lung volume reduced significantly, which made the lung of ARDS appear “baby lung”. This concept was proposed by Gattinoni and Pesenti that refers to the small amount of normally aerated lung units (18). This change is not homogeneous, which is proved by chest computed tomography (CT) scan (19). Due to a small number of aerated lung units, the compliance of the respiratory system decreased. Besides, as lung injury is heterogeneous, some lung regions are better ventilated than perfused, whereas others are less ventilated than perfused or non-ventilated at all (referred to as intrapulmonary shunt). If there is no perfusion because of microcirculatory occlusion, this is referred to dead space. Therefore, there is an imbalance in lung ventilation and perfusion, another important characteristic of ARDS. These changes finally lead to severe hypoxemia without significant improvement by increasing the fraction of inspiration oxygen (20). The level of carbon dioxide may increase due to decreased lung volume and dead space, which can be corrected by an increase in alveolar ventilation (20, 21). Additionally, the characteristics make patients with ARDS prone to ventilator-associated lung injury during ventilator therapy, such as barotrauma, volutrauma and atelectrauma, further aggravate lung injury. What's more, the cardiac output will decrease because of a decrease in the left ventricular preload and an increase in right ventricular afterload up to acute cor pulmonale (21–24).

### Pathophysiology of COVID-19

The pathophysiology of COVID-19 is more complex and diverse comparing to typical ARDS. Some severe COVID-19 patients progressed to classical ARDS, but a considerable proportion of severe COVID-19 cases who progressed to respiratory failure do not conform to the classical ARDS characteristics of “reduced lung volume and decreased compliance”. Of them, the pulmonary compliance is close to normal, which is not consistent with the severity of hypoxemia (25). Moreover, ARDS related hypoxemia is mainly caused by intrapulmonary shunt, supplemented by dead space ventilation (26). COVID-19 related hypoxemia may could be explained by hypoxic pulmonary vasoconstriction dysfunction, resulting in the loss of lung perfusion regulation, and pulmonary vascular microthrombus, which leads to the increase of dead space and intrapulmonary shunt at the same time (7, 27). Graselli et al. observed 301 COVID-19 patients with ARDS and found that the level of D-dimer increased significantly. A higher level of D-dimer was associated with a higher dead space ventilation and a higher mortality (56% with high D-dimers and low compliance vs. 27% with low D-dimers and high compliance, 22% with low D-dimers and low compliance, and 35% with high D-dimers and high compliance, all *p* = 0.0001). In the subgroup of patients who received computed tomography angiography (CTA), there was evidence of bilateral lung hypoperfusion in patients with higher D-dimer levels than the median (28). These data indicate that vascular impairment was existed in COVID-19, and both dead space and intrapulmonary shunt are of important reasons of hypoxemia in COVID-19 patients.

The phenotype of COVID-19 was divided into type H and type L by Gattinoni et al. (25). The main manifestations of L-type were as follows: 1) Low elastance, the nearly normal compliance indicates that the amount of gas in the lung is nearly normal. 2) Low ventilation to perfusion ratio. 3) Low lung weight. 4) Low lung recruitability. In this type of patients, there is severe hypoxemia, but respiratory compliance is more than 50 ml/cm H_2_O.The lung has a large gas content and little recruitability. Severe hypoxemia is mainly due to the imbalance of ventilation and perfusion ratio. At that time, the implementation of high PEEP and prone position does not improve oxygenation by promoting the re-expansion of the collapsed alveolar area, but through the redistribution of pulmonary perfusion, thus improving the ventilation and perfusion ratio. CT scans of both lungs can confirm that there is no obvious recruitable alveolar area in these patients, but the right-to-left shunt venous blood mixture is particularly pronounced about 50% (8). The main manifestations of H-type COVID-19 were as follows:1) Aggravation of pulmonary edema and decrease of lung gas volume, resulting in increase of elastic resistance and decrease of lung compliance. 2) High right-to-left shunt caused by alveolar collapse in gravity-dependent regions. 3) High lung weight. 4) High lung recruitability. H-type is similar to the classical severe ARDS. Professor Maiolo have found that 20–30% of COVID-19 patients admitted to ICU have severe hypoxemia with lung compliance <4 ml/cmH_2_O, suggesting the presence of severe ARDS (29, 30). Of course, their low compliance may be caused by the natural evolution of the disease itself, or it may be related to the initial respiratory therapy. In fact, some patients with severe hypoxemia have received non-invasive ventilation before admission to ICU, and these patients also have strong spontaneous inspiratory efforts and great negative pressure in the chest. Therefore, in addition to viral infection, these patients also have patient self-induced lung injury (P-SILI) (31). Most of the patients with COVID-19 showed L-type in the early stage. With the evolution of the disease and lung injury caused by high stress ventilation, L-type can be transformed into H-type in the late stage.

The most difference in pathophysiology between the typical ARDS and COVID-19 induced ARDS is L-type of COVID-19, which is dramatically different from that of typical ARDS. Several potential explanations may attribute to it. First is the etiology, typical ARDS is always resulted by sepsis, shock, trauma, transfusion and so on, these insults will cause damage to both the endothelium and epithelium, and disruption of the gas and blood barrier, the permeability will increase and large amount of protein-rich fluid flood to the alveolar and interstitium, which lead to reduced lung volume, low compliance and mismatched ventilation and perfusion ratio (9, 20). However, the pathogen of COVID-19 is clear, caused by SARS-CoV-2 and its variants, which mainly attack the endothelium through ACE2 receptor, while the damage to the epithelial cell is mild. Thus, the lung volume and lung compliance are almost normal. Besides, the injured pulmonary endothelium of COVID-19 loss the function of hypoxic vasoconstriction, resulting in the loss of lung perfusion regulation, finally cause the intrapulmonary shunt (7). In addition, the injured pulmonary endothelium of COVID-19 shifts from a normal anti-inflammatory state to an “activated” phenotype characterized by pro-adhesive properties, the production of inflammatory mediators and microthrombus formation, which leads to the increase of dead space (27).

## Clinical Features of COVID-19 and Typical ARDS

### Clinical Manifestation

ARDS is an acute clinical syndrome, often developed within 7 days after attacking by sepsis, shock, trauma, blood transfusion and so on. The most common symptoms of ARDS are respiratory distress and hypoxemia. Sometimes also accompanying other symptoms like fever, cough and so on, which depends on the underlying causes. Patients with COVID-19 always experienced an incubation period of 8 to12 days. The manifestations of COVID-19 are very heterogeneous, they vary from asymptomatic and mild clinical symptoms to severe acute respiratory-distress syndrome (ARDS) and death. Among the severe COVID-19 patients who should be hospitalized, fever (88.7%), cough (67.8%), and dyspnea (63.5%) were the most common symptoms. COVID-19 patients often have symptoms of extrapulmonary organs, such as gastrointestinal symptoms like nausea, vomiting and diarrhea, loss of taste and smell, headache, muscle aches, bone pain and so on (5, 32, 33).

Compared to typical ARDS, some critically ill COVID-19 patients do not present obvious dyspnea even though they have severe hypoxemia, oxygen saturation is lower than 70% and partial pressure of arterial oxygen is lower than 40 mmHg, which is clinically called “silent hypoxemia” or “happy hypoxemia”. The possible mechanisms may be related to poor response to hypoxia, which is influenced by age, medications, coexisting disease and genetic background, abnormal chemoreceptor function of carotid body due to virus attacks, prevailing partial pressure of carbon dioxide inhibit the brain's response to hypoxia, inaccuracy of pulse oximetry at low oxygen saturations, temperature-induced shifts in the oxygen dissociation curve, and different tolerance of low oxygen levels of individuals (34). Cardiorespiratory compensation to hypoxemia is another explanation of “silent hypoxia”. The normal responses are tachycardia and increased cardiac output, however, this response would be limited by age, genetics, and coexisting disease. Failure to compensate for decreased oxygen transport is signaled by lactic acidosis, bradycardia, and decreased cardiac output. The latter may develop rapidly, and all are indicators of impending tissue injury or death from hypoxemia (6).

Both the COVID-19 caused ARDS and the typical one are stratified based on the degree of hypoxemia although there are minor differences between them. According to the Berlin definition, patients with ARDS are divided into mild (200 mmHg ≤ PaO_2_/FiO_2_ <300 mmHg), moderate (100 mmHg ≤ PaO_2_/FiO_2_ <200 mmHg), severe (PaO_2_/FiO_2_ <100 mmHg) at a PEEP level over 5cmH_2_O (3). While patients with COVID-19 pneumonia was classified as mild (200mmHg ≤ PaO_2_/FiO_2_ <300 mmHg), mild to moderate (150mmHg ≤ PaO_2_/FiO_2_ <200mmHg), moderate to severe (PaO_2_/FiO_2_ <150 mmHg) (35).

### Chest Imaging

The radiographic feature of ARDS is bilateral opacities, which was not fully explained by effusions, lobar/lung collapse, or nodules (3). Patients with COVID-19 often have unique imaging manifestations. In the early stage, imaging shows multiple small patchy shadows and interstitial changes, more apparent in the peripheral zone of lungs. As the disease progresses, imaging shows multiple ground glass opacities and infiltration in both lungs. In severe cases, the range of opacities increased and pulmonary consolidation may occur, accompanied by “aerated bronchial sign”. Some severe patients may present with “paving stone sign” and “white lung”, However, pleural effusion is rare. In the late stage, the lung imaging of COVID-19 is similar to that of ARDS (1, 36).

### Laboratory Examination

Abnormal blood gas analysis is the main feature of ARDS patients. The patient always displays low oxygen partial pressure with or without elevated partial pressure of carbon dioxide and lactic acid, sometimes the partial pressure of carbon dioxide will decrease when the patient has respiratory distress. the results of other routine laboratory examination vary greatly due to different etiology. However, as an uncontrolled inflammatory response is the main pathophysiology of ARDS, some patients present systemic inflammatory response syndrome and other organ dysfunction (9).

In addition to the hypoxemia of COVID-19 patients, more and more studies have confirmed that COVID-19 patients tend to show declined lymphocyte, absolute number of T lymphocytes, CD4^+^T and CD8^+^ T cells decreased in nearly all the patients, and were markedly lower in severe cases. Laboratory tests of some COVID-19 patients also showed thrombocytopenia, increased DD dimer, decreased hemoglobin, increased ferritin and C-reactive protein, and increased alanine aminotransferase, aspartate aminotransferase, myocardial enzyme, and increased urea nitrogen (2, 4, 36), suggesting that the patients were in critical condition. Many researchers believed that the inflammatory cytokine storm is closely associated with the development and progression of COVID-19 (37). The expressions of inflammatory cytokines interleukin (IL)-2R, IL-6, IL-10, and TNF-α were significantly increased in COVID-19 patients (36, 37), it was also found that the expressions of some inflammatory mediators were relatively lower in patients with ARDS caused by bacterial infection (38).

## Treatment of COVID-19 and Typical ARDS

### Respiratory Support for ARDS

With the development of ARDS for more than 50 years, the treatment of ARDS is mainly limited to the control of primary etiology and organ support, especially respiratory support. The treatments implemented to patients with ARDS are based on the severity of respiratory distress. For patients with mild ARDS, high-flow nasal catheter oxygen (HFNC) inhalation or non-invasive ventilator assisted ventilation can be given. If oxygenation cannot be maintained or the patient present strong respiratory effort, invasive mechanical ventilation with sedation should be initiated. Lung protective ventilation with a low tidal volume about 6 ml/kg of predicted body weight is the standard treatment for ARDS. Plateau pressure should be monitored continuously and should not exceed 30 cm H_2_O to reduce mortality. For patients with moderate or severe ARDS, high PEEP level should probably be used, and reserved for patients in whom it improves oxygenation with a plateau pressure below 30 cm H_2_O, without marked deterioration of respiratory system compliance or hemodynamic status. If patients' oxygenation does not improve and PaO_2_/FiO_2_ ratio below 150 mmHg after optimizing the ventilator setting, a combination of neuromuscular blockers or prolonged prone ventilation may be used. Neuromuscular blockers are recommended to be administered by continuous infusion early (within the first 48 h of ARDS diagnosis) for no more than 48 h. Prone positioning should be used for at least 16 consecutive hours. Extracorporeal membrane oxygenation (ECMO) should be probably considered in case of severe ARDS patients with PaO_2_/FiO_2_ ratio below 80 mmHg or when mechanical ventilation becomes dangerous because of the increase in plateau pressure and despite optimization of ARDS management including high PEEP, neuromuscular blocking agents, and prone positioning. The decision to use ECMO should be evaluated early by means of contact with an expert center (39). The main principle of treatment is to avoid or reduce ventilator-related lung injury as much as possible while maintaining oxygenation.

### Respiratory Support for COVID-19

In the absence of any clinically proven treatment strategy, the management of COVID-19 is mostly supportive. For ARDS, HFNC inhalation is only suitable for patients with mild ARDS, while HFNC is applicable to a wider range of patients with COVID-19. It is not only suitable for patients with mild ARDS, but also can maintain relatively stable oxygenation and be safe for some patients with moderate and severe ARDS (40). Therefore, for adults with COVID-19 and acute hypoxemic respiratory failure despite conventional oxygen therapy, HFNC inhalation is recommended over conventional oxygen therapy and non-invasive positive pressure ventilation, to maintain patients' peripheral oxygen saturation between 92 to 96% (41). Although there are a lot of differences in the pathogenesis of COVID-19 patients and classical ARDS patients, the current guidelines recommend the use of ARDS therapeutic strategies for respiratory support of COVID-19 patients, due to a high proportion of patients presenting classical clinical and respiratory mechanical characteristics of ARDS. These treatments include low tidal volume ventilation (4–8 ml/kg of predicted body weight) and maintain plateau pressure below 30 cm H_2_O. For mechanically ventilated COVID-19 patients with moderate to severe ARDS and refractory hypoxemia despite optimizing ventilation, selectively use of high PEEP levels, 12 to 16 h of prone ventilation, neuromuscular blockers, recruitment maneuvers (incremental PEEP), or VV-ECMO are recommended (41). For COVID-19 patients without typical ARDS, individualized treatment should be carried out according to the phenotype. For example, for L-type COVID-19 patients, a larger tidal volume (7–8 ml/kg) can be considered due to better lung compliance, and a lower PEEP level (8–10 cm H_2_O) can be used due to low recruitability, in order to reduce ventilator-related lung injury and the adverse effect on hemodynamics (25). Prone position ventilation significantly improves the prognosis of moderate and severe acute respiratory distress syndrome (ARDS) caused by COVID-19, which has been confirmed by many studies (42). From the clinical treatment of COVID-19, 85.7% of intubated patients received prone position ventilation, and 46.2% of patients on noninvasive ventilation (NIV) also tried awake prone position ventilation (43). Most of the COVID-19 patients who received awake prone position ventilation were moderate ARDS patients. It can be seen that oxygenation can be significantly improved after awake prone position ventilation 10 min and most patients have a high tolerance to awake prone position ventilation, 15% of patients say they can accept it, 41% of patients feel good, and 26% of patients feel very good subjective feeling (44). NIV combined with awake prone position ventilation is a bright spot in the treatment of COVID-19. This low-cost but efficient respiratory support strategy can improve patient oxygenation, avoid disease progression and improve prognosis in the short term. However, the effective improvement of oxygenation in COVID-19 hypoxemia after exerting awake prone position may be attributed to the relative homogeneity of lung lesions and mild consolidation; secondly, the degree of hypoxia, NIV compliance, lung consolidation, even age and systemic condition will affect the effect of NIV plus awake prone position ventilation.

### Pharmacological Therapy for Classical ARDS

As ARDS is a clinical syndrome with different etiologies, there is no uniform treatment targeting the underlying causes, which is always individualized, such as the antibiotics are recommended for sepsis and pneumonia. Other pharmacotherapies has focused on: (1) Reducing lung inflammation (methylprednisolone, lisofylline, N-acetylcysteine, macrolides, simvastatin, and neutrophil elastase inhibitors). (2) Reducing interstitial/alveolar edema (surfactant, β2-adrenergic agonists, fluids). (3) Promoting pulmonary vasodilation (nitric oxide and prostacyclin). (4) Acting on coagulation pathways (aspirin, platelet-activating factor receptor antagonists). (5) Improving epithelial (keratinocyte growth factor), endothelial (manipulation of intercellular adhesive junctions), and extracellular matrix repair. A lot of preclinical studies have shown benefits, but clinical studies of N-Acetylcysteine, surfactant, nitric oxide and prostacyclin showed improvement in oxygenation (45). To date, no drug has been able to reduce ARDS morbidity and mortality. Only a secondary analysis of HARP-2 showed that simvastatin was associated to improved survival in those patients with ARDS hyperinflammatory phenotype (46). The results also need more randomized clinical trials to confirm.

### Pharmacological Therapy for COVID-19

COVID-19 is a new disease with clear pathogen, the evidences about the pharmacological therapy for COVID-19 are still limited. Alternate treatment strategies mainly focus on antiviral treatment, anti-inflammatory therapy and anticoagulation, based on the pathogenesis of COVID-19.

#### Antiviral Therapy

In theory, antiviral therapy is supposed to be one of the most important weapons against Covid-19. Antiviral drugs target specific proteins essential for the viral life cycle and disrupt various stages of viral growth. The drugs that can act on a coronavirus can be categorized based on their mechanisms of action (47): (1) Drugs that act on viral proteins and enzymes thus preventing RNA replication and synthesis. (2) Drugs that act on the viral structural proteins, inhibiting self-assembly or blocking the virus from binding to ACE2. (3) Drugs that act on virulence factors and can facilitate the restoration of the host's innate immunity. (4) Drugs that can act on human enzymes or receptors thus blocking viral entry. Few drugs, however, are being developed to target the membrane, nucleocapsid or envelope proteins (48). Among the repurposed candidate drugs like arbidol, chloroquine, camostat, remdesivir, lopinavir, darunavir, ribavirin, favipiravir, galidesivir, oseltamivir, and famotidine (49, 50). Several clinical trials are also underway to evaluate their suitability and efficacy. Remdesivir is the most promising. Remdesivir is a prodrug of a nucleotide analog that is intracellularly metabolized to an analog of adenosine triphosphate that inhibits viral RNA polymerases (51). In a mouse model of SARS-CoV, remdesivir was observed to reduce the lung viral load and improve pulmonary function (52). In the WHO-led, open-label, randomized SOLIDARITY trial, there was a trend toward reduced mortality with remdesivir among patients requiring low-flow or high-flow oxygen at baseline, but not among those requiring mechanical ventilation at baseline, albeit without reaching statistical significance (12.2% in the remdesivir group vs. 13.8% in the control group; RR 0.85, 95% CI 0.66–1.09) (53). In the final results of a large randomized, placebo-controlled trial (ACTT-1 study), patients who received remdesivir had a shorter time to recovery (the primary end point) than those who received placebo [median, 10 days vs. 15 days; rate ratio for recovery, 1.29 (95% CI, 1.12 to 1.49)] and were more likely to have improvement in the ordinal scale score at day 15 (key secondary end point; odds ratio, 1.5; 95% CI, 1.2 to 1.9). Its efficacy was strongest in critically ill patients who needed oxygen early but had not yet been intubated. Unfortunately, the trial did not demonstrate the efficacy of remdesivir in patients who already required mechanical ventilation to initiate treatment. In subgroup analyses, the reduction in time to recovery was only statistically significant in patients randomly assigned 10 days or fewer from symptom onset. Besides, there was a tendency to decrease the all-cause mortality by using remdesivir (11.4% in the remdesivir group vs. 15.2% in the placebo group, hazard ratio, 0.73; 95% CI, 0.52 to 1.03). Among the subset of patients in the ACTT-1 trial requiring oxygen supplementation but not high-flow oxygen or ventilatory support, remdesivir had a significant mortality benefit (4.0% in the remdesivir group vs. 12.7% in the control group; HR 0.30, 95% CI 0.14–0.64) (54). There were similarly results in a prematurely terminated trial of remdesivir in China, patients with COVID-19 and symptom duration of 10 days or less who received remdesivir clinically improved faster than did those who received placebo (HR 1.52, 95% CI 0.95–2.43) (55). Surviving Sepsis Campaign Guidelines on the Management of Adults with Coronavirus Disease 2019 (COVID-19) in the ICU recommend that remdesivir be given intravenously in severe cases of COVID-19 that do not require mechanical ventilation (weak recommendation).

With the emergence of new SARS-CoV-2 genetic variants. The transmissibility increased while the virulence decreased. Many people infected with SARS-CoV-2 are asymptomatic or only with mild symptoms. So, some oral administered antiviral agents such as nirmatrelvir-ritonavir and molnupiravir were tested in non-hospital patients. Nirmatrelvir is an orally administered severe acute respiratory syndrome coronavirus 2 main protease (Mpro) inhibitor with potent pan–human-coronavirus activity *in vitro*. Two trials (EPIC-SR and EPIC-HR) included 3,100 participants with non-severe illness in outpatient settings received nirmatrelvir plus ritonavir, the results showed that nirmatrelvir-ritonavir likely reduces admission to hospital in highest risk patents with non-severe COVID-19 (56). The Guideline Development Group remarked that nirmatrelvir-ritonavir may represent a superior choice because it may have greater efficacy in preventing hospitalization than the alternatives, has fewer concerns with respect to harms than does molnupiravir, and is easier to administer than intravenous remdesivir and the monoclonal antibodies. Thus, the latest version of WHO Life guidelines recommends the use of nimarovir-ritonavir for patients with non-serious diseases with the highest risk of hospitalization. It is conditionally recommended not to use nimarovir-ritonavir in patients with non-serious diseases with a low risk of hospitalization. No recommendations have been made for patients with serious or critical illnesses as there is no data on nirmatrevir-ritonavir in this population. The Guideline Development Group also suggests treatment with remdesivir in patients with non-severe illness at highest risk of hospitalization (57). This updates a previous conditional recommendation against remdesivir made in November 2020.

#### Anti-inflammatory Therapy

Based on current research evidence, the use of corticosteroids in ARDS patients is still controversial and it is not recommended for the treatment of ARDS. However, dexamethasone is the first drug to be demonstrated to improve patient mortality in patients with severe COVID-19. The RECOVERY trial, a large multi-center clinical study conducted in the UK, found that dexamethasone at a dose of 6 mg/ day used for 10 days in severe COVID-19 patients on mechanical ventilation, reduced mortality by 1/3 compared with the control group, but had no significant benefit for patients with mild disease who did not require respiratory support (58). A meta-analysis of systemic glucocorticoids usage in the COVID-19 patients found that administration of systemic glucocorticoids was associated with lower 28-day all-cause mortality, in the included studies, daily glucocorticoid equivalent doses ranged from 6 mg to 20 mg of dexamethasone. The treatment effects were similar with low-dose vs. high-dose regimens (59). Therefore, the guidelines on the management of adult patients with COVID-19 in the ICU recommend that short-term systemic use of glucocorticoids is strongly recommended for severe or critically ill COVID-19 patients requiring supplemental oxygen or mechanical ventilation, with dexamethasone preferred, and other corticosteroids equivalent to 6 mg/day dexamethasone may be considered if dexamethasone is unavailable (41). Until recently, the COVID STEROID 2 Trial Group (60) reports the results of an international, multicenter randomized clinical trial that compared 2 alternative doses, either 12 mg/d or 6 mg/d of dexamethasone in critically ill patients with COVID-19 who were receiving supplemental oxygen at a flow rate of at least 10 L/min or mechanical ventilation. The median number of days alive without life support was 22.0 days (IQR, 6–28 days) in the 12 mg of dexamethasone group and 20.5 days (IQR, 4–28 days) in the 6 mg of dexamethasone group (adjusted mean difference, 1.3 days [95% CI, 0–2.6 days]; *P* = 0.07). Mortality at 28 days after randomization was 27.1% for patients in the 12 mg/d group and 32.3% for patients in the 6 mg/d group (adjusted relative risk, 0.86 [99% CI, 0.68–1.08]). This study suggested that the outcomes for COVID-19 may be further improved by the use of higher doses of glucocorticoids. However, additional trials are needed to confirm this and determine what dose is optimal.

Beside corticosteroid, other drugs targeted the cytokine signaling pathway like interleukin-6 (IL-6) receptor blockers, Janus kinase (JAK) inhibitor were also used in the treatment of COVID-19 patients. JAK inhibitors are a class of drugs which inhibit intracellular signaling through multifactorial effects on cytokine signaling. As a consequence, they interfere with many cellular responses, including antiviral responses, angiotensin-converting enzyme 2 (ACE2) expression, T cell function and differentiation, and macrophage activation (61). Baricitinib, ruxolitinib, and tofacitinib are three of at least nine JAK inhibitors. These three drugs are all generally considered to be non-specific JAK inhibitors. The living network meta-analysis for baricitinib included three randomized clinical trials which enrolled 2,659 patents across disease severities. The results showed that baricitinib probably reduces mortality, duration of mechanical ventilation, hospital length of stay and time to clinical stability in patients with severe and critical COVID-19 (62–64). The effects of ruxolitinib or tofacitinib on mortality, need for mechanical ventilation and hospital length of stay remain uncertain. IL-6 receptor blocker (tocilizumab or sarilumab) is another kind of immune modulator with overlapping effects with baricitinib on immune responses. Accumulating evidences of IL-6 receptor blockers which were evaluated in more than 10,000 participants with severe or critical COVID-19 showed that IL-6 receptor blockers could reduce mortality and need for mechanical ventilation and may also reduce duration of mechanical ventilation and hospitalization (65, 66). Based on these evidences, the latest WHO guideline strongly recommended treatment with IL-6 receptor blockers (tocilizumab or sarilumab) for patients with severe or critical COVID-19 and should be combined with corticosteroids. Baricitinib, is also recommended as an alternative of IL-6 receptor blockers for the treatment of patients with severe and critical COVID-19. The choice of whether to use baricitinib or an IL-6 receptor blocker depends on availability as well as clinical and contextual factors (57).

#### Anticoagulant Treatment

Pathological findings showed extensive microthrombus in pulmonary capillaries of COVID-19 patients. Endothelial injury, cytokine storm and complement cascade hyperactivation probably play an important role in the hypercoagulable state of COVID-19 patients (67). Besides, hospitalized COVID-19 patients are prone to have venous thromboembolism (27, 68, 69), thus, anticoagulation therapy has received great attention from clinicians and researchers, and anticoagulation was suggested as a mitigating option. So far, only retrospective observational studies have shown that anticoagulant therapy improves patient outcomes (70, 71). Tang et al. described 449 patients with severe COVID-19 infection and reported reduced mortality with anticoagulation in patients with sepsis-induced coagulopathy (SIC) score ≥4 (40.0% vs. 64.2%, *P* = 0.029), or D-dimer > 6 fold of upper limit of normal (32.8% vs. 52.4%, *P* = 0.017) (70). It is worth noting that the majority of patients received prophylactic-dose enoxaparin in this study. Which suggested that the prophylactic-dose of anticoagulant is not adequate. Another study from New York examined the effect of therapeutic-dose anticoagulation in 2,773 hospitalized patients with COVID-19. The results showed modest improvement of median survival with the use of anticoagulation. However, this benefit appears to be more significant in mechanically ventilated patients with a 33.6% reduction of mortality (71). There are no randomized controlled trials evaluating the effectiveness of prophylactic-dose of anticoagulant in the COVID-19 population, but evidence from critically ill patients suggests that prophylactic-dose of anticoagulant can benefit critically ill patients in the ICU. The guidelines therefore strongly recommend the use of medications to prevent deep venous thrombosis in adults with severe/critical COVID-19, and do not recommend the use of therapeutic doses of anticoagulants in patients without evidence of thrombosis (41). The optimal anticoagulation agent and dose remain uncertain. Randomized clinical trials are needed to identify the anticoagulation benefit and specify the most effective agent and appropriate dosage.

## Conclusion

When compared with the classical ARDS, COVID-19 has unique pathological and pathophysiological features, with typical pathophysiological changes such as endothelial injury and extensive microthrombus. The coexistence of shunt and dead space ventilation is an important mechanism of hypoxemia in COVID-19 patients. The clinical manifestation of COVID-19 patients is heterogeneous and can present with different phenotypes, with patients presenting with progressive respiratory distress or unique “silent hypoxemia”. Some patients may have the “H-type” characteristics of reduced lung volume, decreased lung compliance, and unmatched ventilator-perfusion ratio, while some patients may have close to normal lung compliance, that is, “L-type”. Identifying different phenotypes is crucial for clinicians to choose appropriate treatment strategy. Some of these therapeutic and prophylactic agents showed some benefits in selected patients, and some randomized trials are presently under the way. It is anticipated that more effective drugs will be found to reduce the global public health damage unleashed by this virus.

## Author Contributions

SL and XH wrote manuscript the initial draft. RL, YLa, YLe, FZ, and XT participated in the manuscript writing. HH supervised and edited the manuscript. All authors contributed to the article and approved the submitted version.

## Funding

This work was supported by National Natural Science Foundation of China (Grant no. 81700073), Chengdu Science and Technology Project for COVID-19 prevention and control (Grant nos. 2020-YF05-00050-SN and 2020-YF05-00070-SN), and 2020 Major medical innovation project of Sichuan Provincial Health Commission (20ZDCX002).

## Conflict of Interest

The authors declare that the research was conducted in the absence of any commercial or financial relationships that could be construed as a potential conflict of interest.

## Publisher's Note

All claims expressed in this article are solely those of the authors and do not necessarily represent those of their affiliated organizations, or those of the publisher, the editors and the reviewers. Any product that may be evaluated in this article, or claim that may be made by its manufacturer, is not guaranteed or endorsed by the publisher.
